# An MR technique for simultaneous quantitative imaging of water content, conductivity and susceptibility, with application to brain tumours using a 3T hybrid MR-PET scanner

**DOI:** 10.1038/s41598-018-36435-8

**Published:** 2019-01-14

**Authors:** Yupeng Liao, Ana-Maria Oros-Peusquens, Johannes Lindemeyer, Nazim Lechea, Carolin Weiß -Lucas, Karl-Josef Langen, N. Jon Shah

**Affiliations:** 1 Institute of Neuroscience and Medicine-4, Forschungszentrum Jülich, Jülich, Germany; 2 Department of Neurosurgery, University Hospital Cologne, Cologne, Germany; 3 Department of Nuclear Medicine, RWTH Aachen University Clinic, Aachen, Germany; 4 Department of Neurology, RWTH Aachen University Clinic, Aachen, Germany; 5 JARA-Faculty of Medicine, RWTH Aachen University, Aachen, Germany

## Abstract

Approaches for the quantitative mapping of water content, electrical conductivity and susceptibility have been developed independently. The purpose of this study is to develop a method for simultaneously acquiring quantitative water content, electrical conductivity and susceptibility maps based on a 2D multi-echo gradient echo sequence. Another purpose is to investigate the changes in these properties caused by brain tumours. This was done using a 3T hybrid magnetic resonance imaging and positron emission tomography (MR-PET) scanner. Water content maps were derived after performing T_2_* and transmit-receive field bias corrections to magnitude images essentially reflecting only the H_2_O content contrast. Phase evolution during the multi-echo train was used to generate field maps and derive quantitative susceptibility, while the conductivity maps were retrieved from the phase value at zero echo time. Performance of the method is demonstrated on phantoms and two healthy volunteers. In addition, the method was applied to three patients with brain tumours and a comparison to maps obtained from PET using O-(2-[18 F]fluoroethyl)-L-tyrosine and clinical MR images is presented. The combined information of the water content, conductivity and susceptibility may provide additional information about the tissue viability. Future studies can benefit from the evaluation of these contrasts with shortened acquisition times.

## Introduction

Interest in quantitative magnetic resonance imaging for studying various brain pathologies has recently grown due the specificity of the information accessible with this technique. Compared to conventional T_1_-weighed and T_2_/T_2_*-weighted non-quantitative MRI, quantitative MRI provides a reproducible measure of a given physical parameter e.g. cerebral water content and ferritin concentrations, which are linked to various physiological parameters of the brain tissue and have been explored in clinical studies^1–4^. In particular, water content imaging provides information about the MR-visible tissue proton density which is simply termed the “water content”; the fraction of protons, from macromolecule etc., that are not visible are neglected. Further, electrical conductivity and susceptibility mapping describe the electric and magnetic properties of the underlying tissue. In the healthy brain these parameters are highly regulated^5–7^. However, in a variety of pathological conditions, such as oedema, cancer, stroke and neurodegeneration, abnormal alterations have been found. Therefore, efforts have been made to establish a reliable assessment of quantitative MRI images^8–10^.

However, the current protocols used in the aforementioned studies were usually performed separately, even though water content, electric and magnetic properties are internally linked through Maxwell’s equation and Maxwell mixture theory^11^. This use of single contrast acquisitions has several drawbacks: long acquisition times are implicit and, moreover, independently measured images are not naturally co-registered and may thus cause spatial mismatch when performing further analysis. To overcome these drawbacks, a recent study demonstrated the feasibility of simultaneous mapping of susceptibility and conductivity based on the phase profile of a multi-echo gradient echo (GRE) sequence^12^. In particular, quantitative susceptibility values are estimated based on the phase evolution over time, while the electrical conductivity is calculated from the transceiver phase, which is again obtained by interpolating the phase evolution to TE = 0. This method only makes use of phase information from the multi-echo GRE sequence. However, also the magnitude variation over echo time contains useful information. It is directly related to the degree of magnetic field inhomogeneity (effective transverse relaxation rate, R_2_*) and by interpolation to TE = 0 it can be further utilised to retrieve a map of the water content by involving a series of correction and calibration steps.

In this work, a new method for the simultaneous acquisition of water content, susceptibility and conductivity within a single measurement is presented. Due to a novel post-processing technique that fully utilises both the magnitude and phase profile of a 2D long-TR multi-echo GRE sequence, quantitative multi-parametric maps can be acquired on a 3T scanner. The feasibility of the method is demonstrated firstly in phantom experiments and further *in vivo* in two healthy controls. Finally, the potential of the method is explored in three patients with brain tumours using a hybrid 3T MR-PET scanner. Particularly in the patient measurements, the potential value of the combined information of water content and susceptibility, as well as electrical conductivity, can be evaluated in comparison with standard clinical sequences and positron emission tomography (PET) images.

## Theoretical Background

### Water content mapping

To ensure a predominance of water content contrast, an M_0_ (spin density, in proportion to proton density) weighted multi-slice radio frequency (RF)-spoiled GRE MRI sequence with a long repetition time, TR = 10 s, is employed. Since all of the white matter (WM) and 99% of the grey matter (GM) in the brain have T_1_ < 2 s at 3T^13^, T_1_ corrections to the steady state of WM and GM are negligible for TR = 10 s. Therefore, the magnitude signal for the spoiled gradient echo in a voxel with uniform magnetization, M_0_ and uniform relaxation time T_2_* can be described as:

1 $${\rm{s}}({{\rm{M}}}_{0})={{\rm{M}}}_{0}\,\sin ({\alpha }_{actual}){{\rm{B}}}_{1}^{-}{{\rm{e}}}^{-{\rm{TE}}/{{\rm{T}}}_{2}^{\ast }}$$

where M_0_, and TE denote the equilibrium magnetisation density and the echo time respectively, ${{\rm{\alpha }}}_{{\rm{actual}}}={{\rm{\alpha }}}_{{\rm{nom}}}{{\rm{B}}}_{1}^{+}$, where α_actual_ is the actual flip angle and α_nom_ is the nominal one^14^. The term ${{\rm{B}}}_{1}^{+}$ and ${{\rm{B}}}_{1}^{-}\,$denote the transmit and receive sensitivity profiles of the RF coil. The evolution of the magnitude of M_0_-weighted images as a function of the echo time, TE, was first fitted to a mono-exponential model ${\rm{S}}({\rm{TE}})={{\rm{S}}}_{0}{{\rm{e}}}^{-{\rm{TE}}/{{\rm{T}}}_{2}^{\ast }}$, providing an estimate of the T_2_* relaxation time and signal intensity at TE = 0 on a voxel-by-voxel basis. Moreover, due to the multiplicative nature of the transmit-receive field correction factor as described in Equation(1), M_0_ and the inhomogeneity caused by transmit-receive field can be directly estimated using a probabilistic framework as implemented in SPM8 toolbox^15^ (http://www.fil.ion.ucl.ac.uk/spm/software/spm8/).

### Electrical conductivity mapping

The spatial phase distribution of the GRE signal, varying with echo time, can be modelled as:

2 $${\rm{\phi }}({{\rm{r}}}_{0},{\rm{TE}})\sim {\rm{\phi }}({{\rm{r}}}_{0},{{\rm{B}}}_{1}^{+})+{\rm{\phi }}({{\rm{r}}}_{0},{{\rm{B}}}_{1}^{-})+{\rm{\gamma }}\cdot {{\rm{\Delta }}{\rm{B}}}_{0}({{\rm{r}}}_{0})\cdot {\rm{TE}}$$

where ${\rm{\phi }}({{\rm{B}}}_{1}^{+})$ represents the phase of the transmit field during RF excitation, while ${\rm{\phi }}({{\rm{B}}}_{1}^{-})$ denotes the initial receive coil phase offset profile. Furthermore, γ denotes the gyromagnetic ratio and ΔB_0_ represents the variation of the local B_0_ field caused by the distribution of magnetic susceptibility within the subject and its surroundings. Possible time variations of this field, due e.g. to eddy currents or physiological motion, are neglected. The sum of ${\rm{\phi }}({{\rm{B}}}_{1}^{+})$ and ${\rm{\phi }}({{\rm{B}}}_{1}^{-})$, the transceive phase ϕ^±^, remains constant over time. It is determined by factors including electrical properties, the transmit and receive sensitivity of coils and the location of the subject relative to the coils^12^. To extrapolate the transceive phase ϕ^±^ back to TE = 0 and evaluate phase information, a multi-echo GRE sequence is employed.

Although algorithms for quantitative susceptibility mapping (QSM) calculation from phase evolution profiles are commonly used, methods for quantitative conductivity mapping (QCM) are still an active, ongoing research area^16–19^. In this study, the phase-based magnetic resonance electrical properties tomography (MREPT) was applied to calculate the electrical conductivity (σ) using the following equation:

3 $$\sigma ({r}_{0})={\nabla }^{2}\phi ({r}_{0},{B}_{1}^{+})/{\mu }_{0}\omega $$

where, ${\rm{\phi }}({{\rm{B}}}_{1}^{+})$ denotes the phase of transmit field and ω is the Larmor frequency, μ_0_ is the vacuum permeability, and ∇ represents the Laplacian operator. In our study, the RF transmit phase ${\rm{\phi }}({{\rm{B}}}_{1}^{+})$ was estimated as half of the transceiver phase ϕ^±^ value computed at TE = 0^10,17^.

## Methods

### Phantom imaging protocol

We evaluated the methods for simultaneous mapping of water content, conductivity and susceptibility, in phantom measurements. A multi-compartment cylinder water phantom containing 3 tubes with concentrations of (i) 15% D2O, 0.5% gadolinium and 1.8% NaCl; (ii) 25% D2O, 1% gadolinium and 3% NaCl; (iii) 35% D2O, 1.5% gadolinium and 4.5% NaCl was constructed. The D_2_O concentration was varied to modulate H_2_O content; the NaCl concentration was adapted to manipulate the electrical conductivity while the gadolinium (Gd) concentration was changed to control magnetic susceptibility. Note that adding Gd not only adjusted the phantom susceptibility but also decreased the phantom T_1_ drastically. Therefore, when TR = 10 s was applied, a full T_1_ relaxation occurred and no extra T_1_ correction was required for water content mapping of the liquid phantom.

Phantom data were acquired using a 3T Siemens Magnetom Trio scanner. Considering the size of the phantom, a quadrature birdcage head coil was used for both transmission and reception. The 2D long-TR multi-echo GRE scans for the phantom were performed with the following parameters: TR = 10 s; first TE = 4.23 ms; echo spacing ΔTE = 3.79 ms; number of echoes = 28; flip angle = 90°; field-of-view (FOV) = 192 mm × 192 mm; voxel size = 1.0 mm × 1.0 mm; slice thickness = 1.5 mm; number of coronal slices = 60; monopolar readout; scan time = 23 min 55 s. In addition, for phantom measurement where measurement time is not a strict constraint, a fast mapping protocol was employed as an alternative way to perform RF field inhomogeneity correction. This estimation is based on the principle of the reciprocity, where the transmit sensitivity provides a good approximation of the receive sensitivity for a birdcage transmit-receive head coil, the actual flip angle α_actual_ was estimated using a set of single-shot 2D GRE-EPI acquisitions with variable flip angles. Parameters were: flip angles = 30°, 60°, 90°, 120°; TR = 20 s; TE = 12 ms; BW = 2520 Hz/pixel; 2 mm slice thickness. The sequence for mapping the flip angle was adjusted to the same slice orientation and field-of-view as the M_0_-weighted scan. The scan time for acquiring one flip angle map was 20 s.

To evaluate the performance of the proposed 2D GRE based phase processing, additional 3D GRE scans were carried out during the same measurement session. The quantitative conductivity and susceptibility maps obtained from the 2D method were then compared with those reconstructed from the conventional 3D GRE based phase processing. The 3D GRE phantom imaging parameters were as follows: TR = 60 ms; first TE = 1.5 ms; ΔTE = 2.5 ms; flip angle = 14°; number of echoes = 8; voxel size = 1.0 mm isotropic; monopolar readout; scan time = 30 min 50 s.

### Healthy control imaging protocol

*In vivo* scans were carried out on two healthy volunteers (male, aged 27 and 32 years). The healthy volunteers study was approved by the Ethics Committee of the Institute of Neuroscience and Medicine 4, Forschungszentrum Jülich. All the methods were performed in accordance with the relevant guidelines and regulations. Prior written, informed consent was obtained from all subjects before measurements and the study was conducted according to the Declaration of Helsinki.

The MR signal reception for healthy volunteers was performed using a dedicated RF system consisting of a birdcage transmit coil and 8-channel phase array head coil. The 2D long-TR multi-echo imaging parameters for the healthy volunteers were: TR = 10 s; first TE = 3.73 ms; ΔTE = 4.01 ms; number of echoes = 12; flip angle = 90°; field-of-view = 220 mm × 178 mm, voxel size = 1.15 mm × 1.15 mm; slice thickness = 1.5 mm; number of transversal slices = 72; mono polar read out. Parallel imaging was employed with an acceleration factor of two and partial Fourier imaging was used with a factor of 6/8. As a result, scanning time was reduced to 7 min 37 s.

3D GRE protocol was also applied for healthy volunteers. The acquisition parameters were TR = 60 ms; first TE = 2.5 ms; ΔTE = 2.85 ms; flip angle = 15°; number of echoes = 20; voxel size = 1.0 mm isotropic; monopolar readout; parallel acceleration factor = 2; partial Fourier factor = 6/8. scan time = 11 min 54 s.

### MR-PET experiments and imaging protocol

PET, using the amino acid tracer O-(2-[18 F] fluoroethyl)-L-tyrosine (FET)-PET (half-life of 18 F: 109 min), was employed as part of the study. This PET tracer enables a more reliable delineation of tumour tissue, especially in high-grade gliomas, which displays a higher amino acid uptake than normal brain tissue. Therefore, using FET-PET allows for better differentiation of tumour tissue from surrounding oedema compared to conventional MRI^20,21^. During the experiment, PET, as well as MR images, were obtained on a 3T hybrid MR-PET scanner. A birdcage head coil was used for RF transmission and an 8-channel receiver coil for MR signal detection. In this study, for proof-of-principle and testing feasibility of the proposed method, the three most common entities of intracranial tumours (meningioma, glioblastoma and metastasis) were investigated. All patients were treated in the Department of Neurosurgery, Cologne University Hospital, Germany. The patient study was approved by the Ethics Committee of the Medical Faculty of the University of Cologne. The research is in compliance with the Declaration of Helsinki.

For quantitative multi-parametric mapping, a 2D long-TR multi-echo M_0_-weighted GRE sequence was employed with similar parameters as used in healthy volunteers studies. However, to reduce the scanning time, and thus making this sequence a viable add-on to many clinical protocols, parallel imaging and a slightly larger in-plane voxel size were employed. The imaging parameters were as follows: TR = 10 s; first TE = 3.87 ms; ΔTE = 4.08 ms; flip angle = 90°; number of echoes = 12; field-of-view = 220 × 158, voxel size = 1.15 mm × 1.15 mm; slice thickness = 1.5 mm; number of transversal slice = 60; monopolar readout; parallel acceleration factor = 2; partial Fourier factor = 6/8. The acquisition time was 7 min 21 s. Two additional clinical MR protocols were acquired, including MP-RAGE and FLAIR.

### Processing

**Figure 1:**
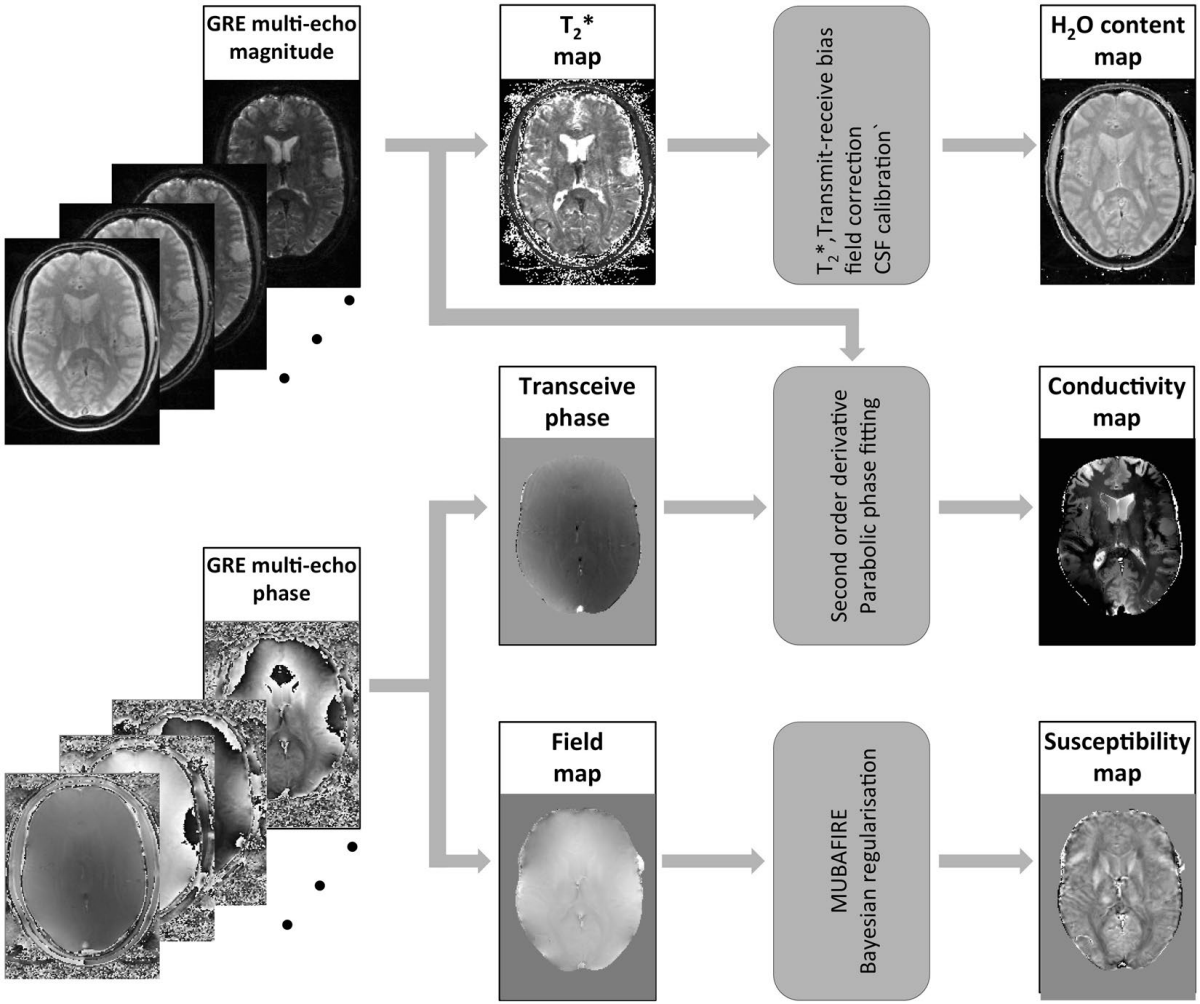
Schematic representation of the quantitative multi-parametric mapping workflow with data from a brain tumour patient: the gradient multi-echo phase is unwrapped and separated into field and transceiver (TE = 0) components, from which susceptibility and conductivity maps are reconstructed. The M_0_-weighted magnitude images are utilised for H_2_O quantification after a series of corrections including T_1_, T_2_* and RF field inhomogeneity bias correction. Moreover, the magnitude profiles additionally serve for the identification of tissue boundaries information required in the reconstruction of conductivity.

Magnitude and phase data were saved for all the echoes acquired from GRE sequences and processed off-line. The procedure of processing magnitude and phase images for patients is illustrated in Fig. 1 as an example. The procedures for each quantitative parameter are detailed below.

### Water content

The magnitude decay with respect to echo time was fitted to a mono-exponential model voxel by voxel, generating maps of the transverse relaxation time T_2_* and signal intensity, S_0,_ at zero echo time. S_0_ is proportional to M_0_, the equilibrium magnetization density. SPM-based bias field correction was performed in healthy controls and tumour patients, while, as already noted, in the phantom experiment, an additional ${{\rm{B}}}_{1}^{+}$ mapping correction method was performed. Following the T_2_* and transmit-receive field non-uniformity correction, the M_0_ signal was normalised to the cerebrospinal fluid (CSF) signal. This then acted as an internal reference which was considered to correspond to 100% water content. Due to the very long repetition time (10 s), T_1_ saturation effects in WM and GM can be neglected at 3T. However, this is not the case for the CSF signal, since its T_1_ is much longer. Thus, compensation for the long T_1_ value was required: based on the measured effective flip angle in the CSF and given a T_1_ value of 4.3 s, a correction factor of 0.94 was used throughout the calculation^8,14,22^.

### Magnetic susceptibility

The QSM processing was done using in house-developed Matlab-based software as follows. To obtain QSM maps from the GRE phase images, two crucial preliminary steps are required: phase unwrapping and background phase removal. All echoes were first spatially unwrapped using an algorithm proposed by Abdul-Rahman^23^. Thereafter, echoes were correctly aligned in the temporal dimension using temporal unwrap of the phase from voxels with very small field inhomogeneity. The field maps were computed by applying a linear regression to the temporal evolution of the unwrapped phase during the echo times. The linear regression also gives the TE = 0 ms phase information, which is used as a starting point in the electrical conductivity processing (see next section). The 2D field maps were interpolated in the through-plane direction in order to provide an isotropic sampling of the volume-of-interest. The contribution of external background fields was then removed using a hybrid approach named MUBAFIRE^24^, separately dealing with spherical harmonic background functions and with dipole fields. The remaining field characteristics are, ideally, generated only by susceptibility contrast from within the brain support. Finally, the dipole inversion was carried out using a Tikhonov- and gradient-regularised minimisation algorithm, resulting in the susceptibility map^25^. The results of the resampled 2D QSM and the isotropic 3D QSM were compared. The CSF value was then chosen as reference value and set to zero.

### Electrical conductivity

The QCM processing was done using in house-developed Matlab-based software as follows. The TE = 0 ms phase information (transceive phase) was obtained as described above for the QSM reconstruction (spatial unwrapping, temporal alignment and linear fit to the phase evolution). Additional care should be taken when choosing the optimal number of echoes for an accurate zero echo time phase estimation. A previous study has shown that for a given a time interval (5.5 ms for instance), the maximum TE used should be roughly equal to the T_2_* value in order to maximise the accuracy of phase estimation^12^. The mean T_2_* value for WM and GM at 3T is around 50 ms with the width of distribution of around 20 ms^13^. In order to maintain the accuracy of the method in regions with shorter T_2_*, in our study, we included in the reconstruction all the echoes with TE around 30 ms. As a result, 8 echoes were effectively used for 2D *in vivo* QCM while 10 echoes were included for 3D *in vivo* QCM.

The electrical conductivity maps were then calculated, essentially as the second order spatial derivative from the estimated zero echo time phase images. To reduce artefacts arising from boundary voxels when computing the Laplacian of the phase, a 3D polynomial fitting method was implemented. During the fitting procedure, neighbouring voxels around each target voxel, r_0_, were first selected to form a spherical fitting kernel (kernel radius = 5 mm). To ensure that the phase information is taken from homogeneous regions, the fitting kernel was further limited to voxels, r, satisfying |S(r)/S(r_0_)−1| < R_thresh_(R_thresh_ = 20%), where S(r) denotes the magnitude of the M_0_-weighed signal^26^. The Laplacian of the target voxel phase was then estimated from the second order coefficients of the fitted polynomial. Following the fitting process described above, additional median filters were applied, with a large spherical kernel (kernel radius = 12 mm) size and were restricted to voxels with R_thresh_ = 20%.

### Segmentation and masks

To calculate the quantitative values in different regions-of-interest (ROIs) for the healthy controls, masks of three tissue classes, WM, GM and CSF, were produced. This was done using the SPM8 package and was based on the anatomy profile ascertained from the magnitude of the first echo from the long-TR GRE acquisition. The averaged values and standard deviations were then obtained from the reconstructed maps for each tissue class. For the patient data, all the relevant data (MPRAGE, FLAIR and PET) were firstly coregistered to the first echo of GRE magnitude images as reference images. Initial masks of WM, GM and CSF were obtained using SPM8 based on the GRE magnitude images. The initial masks were complemented by tumour masks, which were segmented from PET images, and oedema masks, which were obtained from FLAIR images by applying an appropriate threshold. The masks were mutually exclusive. The mean values and standard deviations were then obtained from the reconstructed maps for each mask. Additionally, to investigate the physical changes in abnormal tissue of the brain, a comparison of the parameter values was performed for each of the tissue groups.

## Results

### Phantom and healthy controls

**Figure 2:**
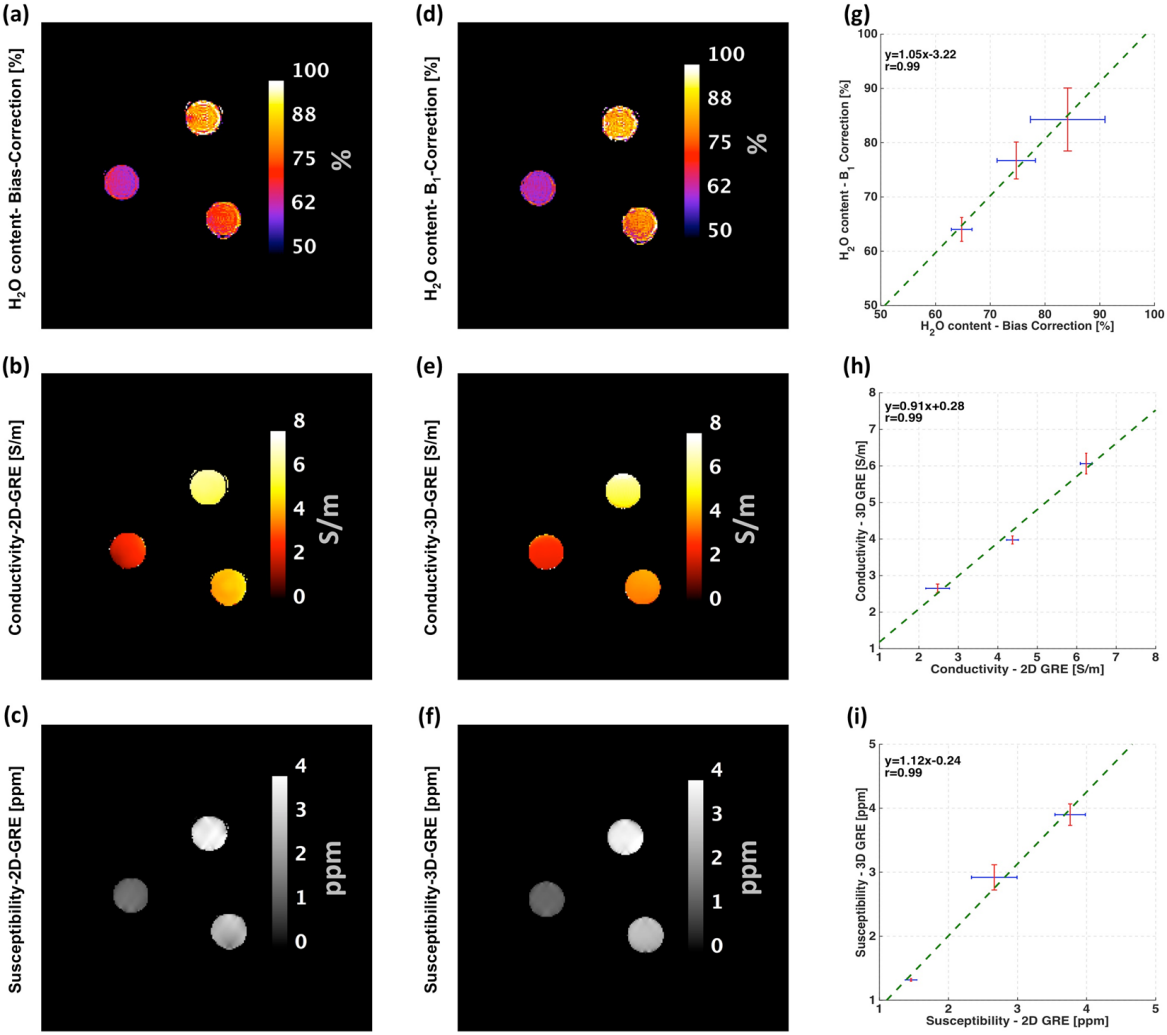
Phantom experiments with varying H_2_O content, susceptibility and conductivity. Part one: phantom H_2_O content obtained from 2D-multi-echo GRE magnitude images using different RF bias correction methods: (**a**) SPM8 based RF bias correction (**d**) B_1_^+^ based RF correlation (**g**) correlation between RF bias correction methods; Part two: Phantom conductivity obtained from 2D and 3D multi-echo GRE phase images: (**b**) 2D method conductivity (**e**) 3D method conductivity (**h**) comparison between 2D and 3D method; Part three: phantom susceptibility calculated from 2D and 3D multi-echo GRE images: (**c**) 2D method susceptibility (**f**) 3D method susceptibility, i) correlation between 2D and 3D method. The length of the error bars represents twice the standard deviation of the parametric map in the ROI.

Maps of the calculated water content, conductivity, and susceptibility parameters of the phantom using the proposed 2D multi-echo GRE sequence method are shown in Fig. 2. The design, as well as measured D_2_O, NaCl and Gd concentrations all increased - in clockwise direction with the lowest concentrations on the left. The quality of the parametric maps was investigated, as shown in Fig. 2a–c, through comparison with maps acquired from reference protocols, including ${{\rm{B}}}_{1}^{+}$ corrected H_2_O maps, 3D-GRE-based QCM and QSM maps (Fig. 2d–f). Three scatter plots (Fig. 2g–i) further illustrate this comparison by showing the measured water content, conductivity and susceptibility value averaged over a region-of-interest in a tube.

**Figure 3:**
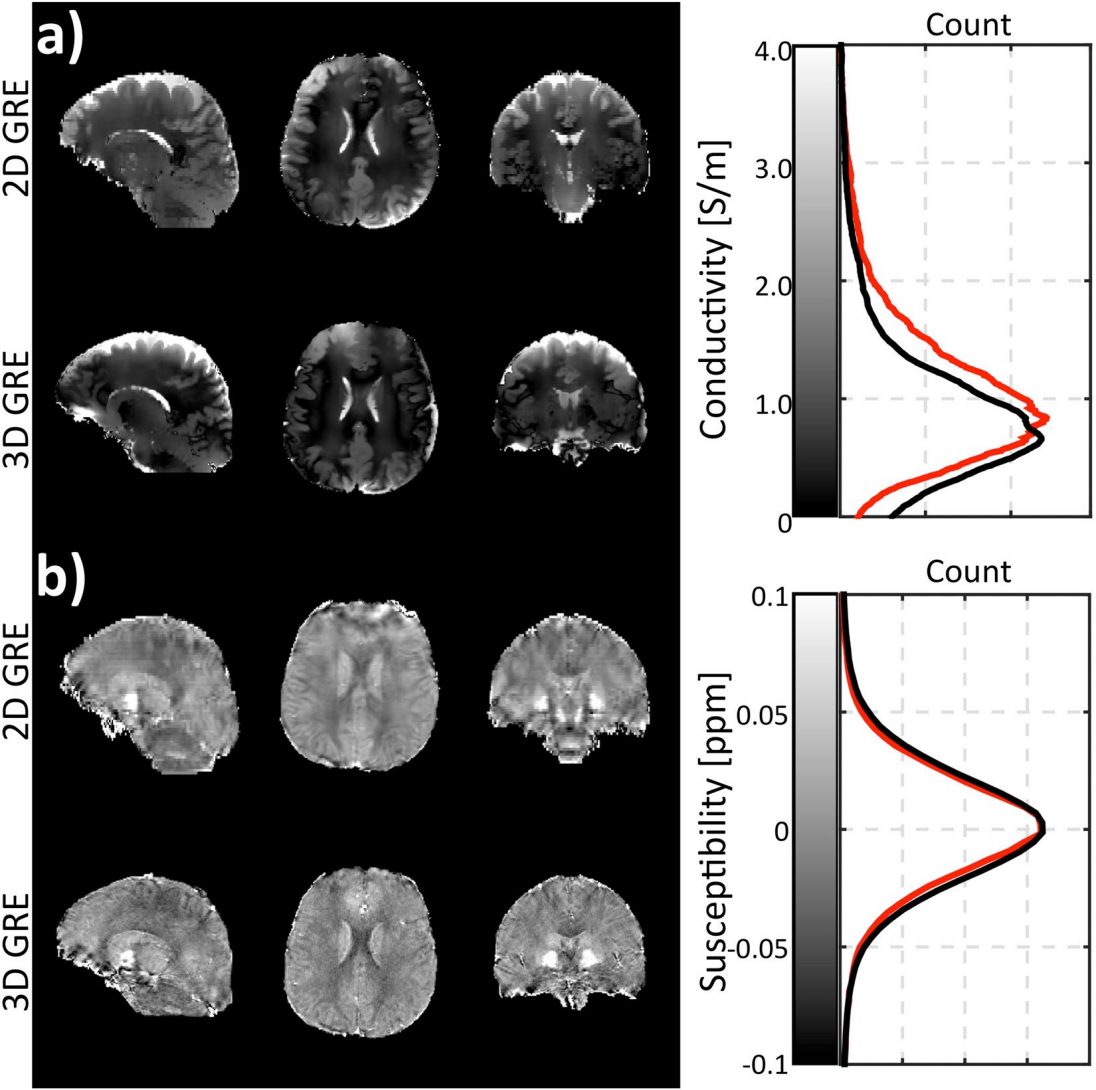
*In vivo* reconstructed images obtained using the proposed method in comparison to the results from a 3D protocol. Sagittal, transverse and coronal slices of the estimated (**a**) susceptibility (**b**) conductivity maps. For each parameter, the first row contains the results of the proposed method, while the second row displays the 3D protocol results. The corresponding histograms are demonstrated on the right of each parametric map. The red line and black line represent the proposed 2D protocol and 3D protocol, respectively.

The reconstructed conductivity and susceptibility maps obtained from the 2D multi-echo GRE and the 3D protocol in one healthy control are displayed in three orthogonal planes in Fig. 3. The associated density plots are shown for both the 3D protocol maps (black line) and the 2D multi echo GRE protocol (red line). These plots represent the empirical distribution of each quantitative parameter for the whole brain volume. ROIs masks, including WM, GM and CSF, were generated and multiplied by the reconstructed 2D multi-echo GRE based H_2_O content, QSM and QCM maps. The average values and standard deviations of each parameter in healthy brain tissue were then assessed and reported in Table 1. We note here that the standard deviation characterises the width of the distribution of the parameters in tissue, and not the accuracy of the method.

**Table 1:** Estimated water content, susceptibility and electrical conductivity values in normal and pathological brain tissue regions^†^.

Brain Regions		Healthy Control		Pathological Brain Tissue		
		Control 1	Control 2	Patient 1	Patient 2	Patient 3
Water Content (%)	White Matter	70.56/5.2	71.10/2.4	69.11/3.5	71.58/2.9	70.14/3.0
	Grey Matter	79.32/6.7	82.30/4.0	83.80/5.2	85.03/3.8	84.46/2.4
	CSF*	100/2.0	100/2.6	100/3.6	100/3.5	100/2.0
	Tumour	N/A	N/A	86.00/6.5	86.20/4.9	81.20/5.7
	Oedema	N/A	N/A	N/A	82.23/7.2	81.14/4.3
Conductivity (S/m)	White Matter	0.57/0.23	0.58/0.22	0.44/0.22	0.66/0.27	0.48/0.23
	Grey Matter	0.93/0.42	1.06/0.56	1.02/0.69	1.07/0.53	0.97/0.44
	CSF	2.30/0.34	1.97/0.54	2.16/0.42	1.905/0.17	1.955/0.23
	Tumour	N/A/	N/A	0.83/0.49	1.15/0.21	1.13/0.55
	Oedema	N/A/	N/A	N/A	1.15/0.46	1.22/0.24
Susceptibility (ppm)**	White Matter	−0.055/0.007	−0.053/0.006	−0.058/0.008	−0.065/0.013	−0.050/0.011
	Grey Matter	−0.020/0.036	−0.025/0.025	−0.024/0.030	−0.028/0.027	−0.019/0.023
	CSF ***	0/0.014	0/0.021	0/0.008	0/0.020	0/0.020
	Tumour	N/A	N/A	−0.01/0.007	−0.052/0.014	−0.067/0.010
	Oedema	N/A	N/A	N/A	−0.047/0.011	−0.046/0.010

^†^Data show mean/standard deviation of the Gaussian distribution fit.

*Normalized value. CSF water content assumed to be 100%.

**Relative value (difference) with respect to CSF.

***Reference value. CSF susceptibility set to 0 ppm.

### Tumour patients imaging

**Figure 4:**
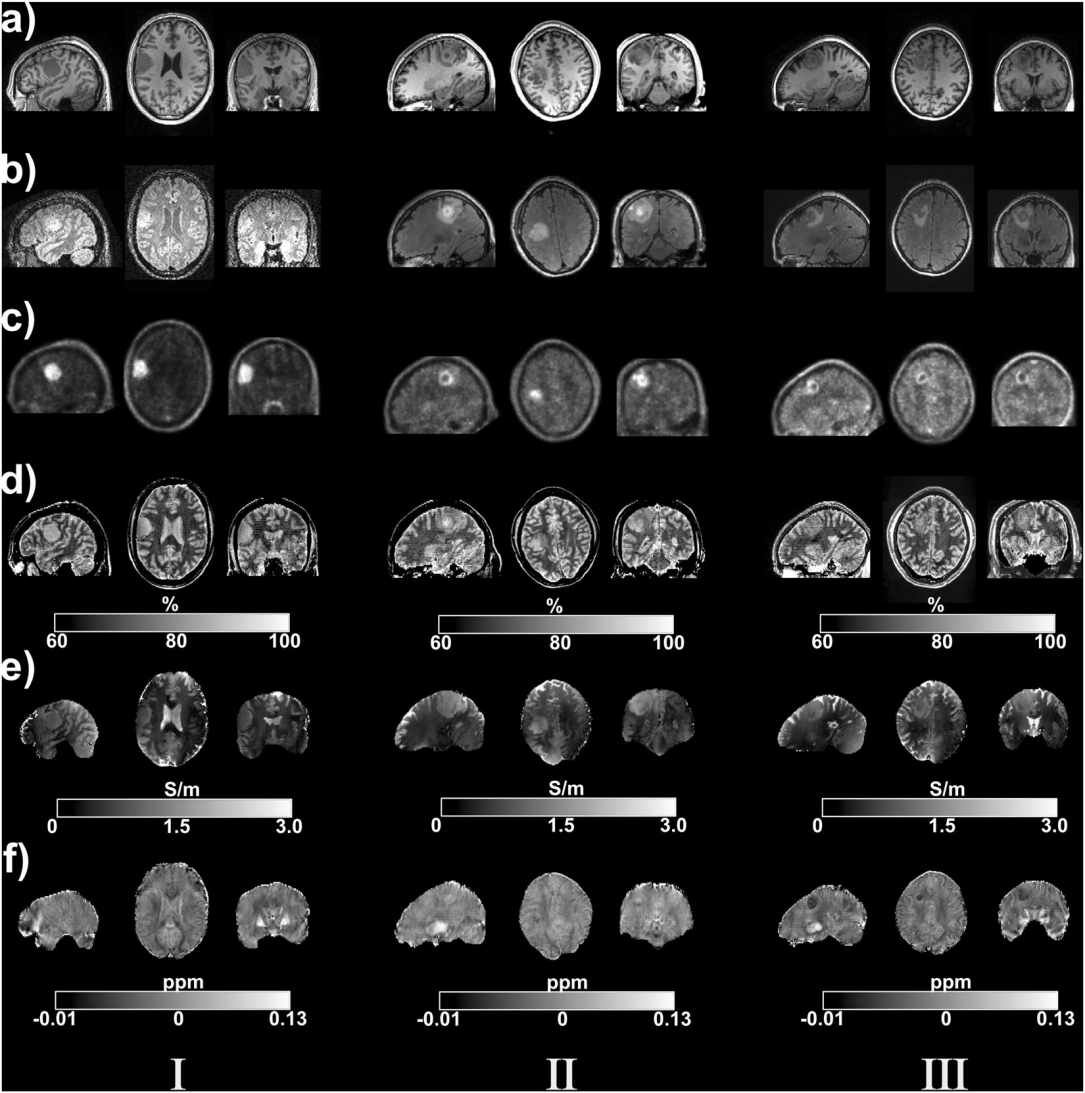
MR and PET images from three tumour patients. Sagittal, transverse and coronal slices through the tumours. (**a**) T_1_-weighted MPRAGE (**b**) proton FLAIR (**c**) FET-PET (**d**) quantitative water content (**e**) electrical conductivity and (**f**) quantitative susceptibility images. The colour bar indicates the water content in percentage, susceptibility in ppm and conductivity in S/m in the images.

Contrasts from multiple modalities obtained from three untreated brain tumour patients are illustrated in sagittal, coronal, and transversal views in Fig. 4. The individual tumour type was determined subsequent to the present study by histology. Tumour I was a meningioma (atypical meningioma WHO II°), tumour II was a glioblastoma (malignant glioma WHO IV°) and tumour III a metastasis (metastasis of a colorectal adenocarcinoma). MR images obtained from clinical MR protocols (including MP-RAGE and FLAIR) and co-registered to the long TR 2D GRE maps are displayed in (Fig. 4a,b). Reconstructed FET-PET maps are shown in (Fig. 4c). Abnormal regions appeared as hypo-intense areas in the MP-RAGE, while hyper-intensity was found in the FLAIR images in the same location. The FET-PET images display a high uptake of amino acid in the tumour regions and demonstrate sharp edges, which enable better delineation of tumour tissue from surrounding tissue and oedema. Simultaneously acquired H_2_O content, QSM and QCM maps from the long TR GRE protocol are illustrated in (Fig. 4d–f), respectively. All quantitative maps acquired from healthy controls and the patient group demonstrated good anatomical contrasts. In patients, the H_2_O content maps revealed large hyper-intense regions, which included tumour and oedema (Fig. 4d), while QSM maps displayed varying contrast. In patient I and patient II (meningioma, glioblastoma), regions which are more paramagnetic than healthy tissue were found; however, in patient III (metastasis), higher (more diamagnetic) susceptibility was found to characterise the abnormal regions (Fig. 4e). Hypo-intensity was observed in abnormal tissues, relative to the healthy contralateral side, in QCM images in all three cases.

**Figure 5:**
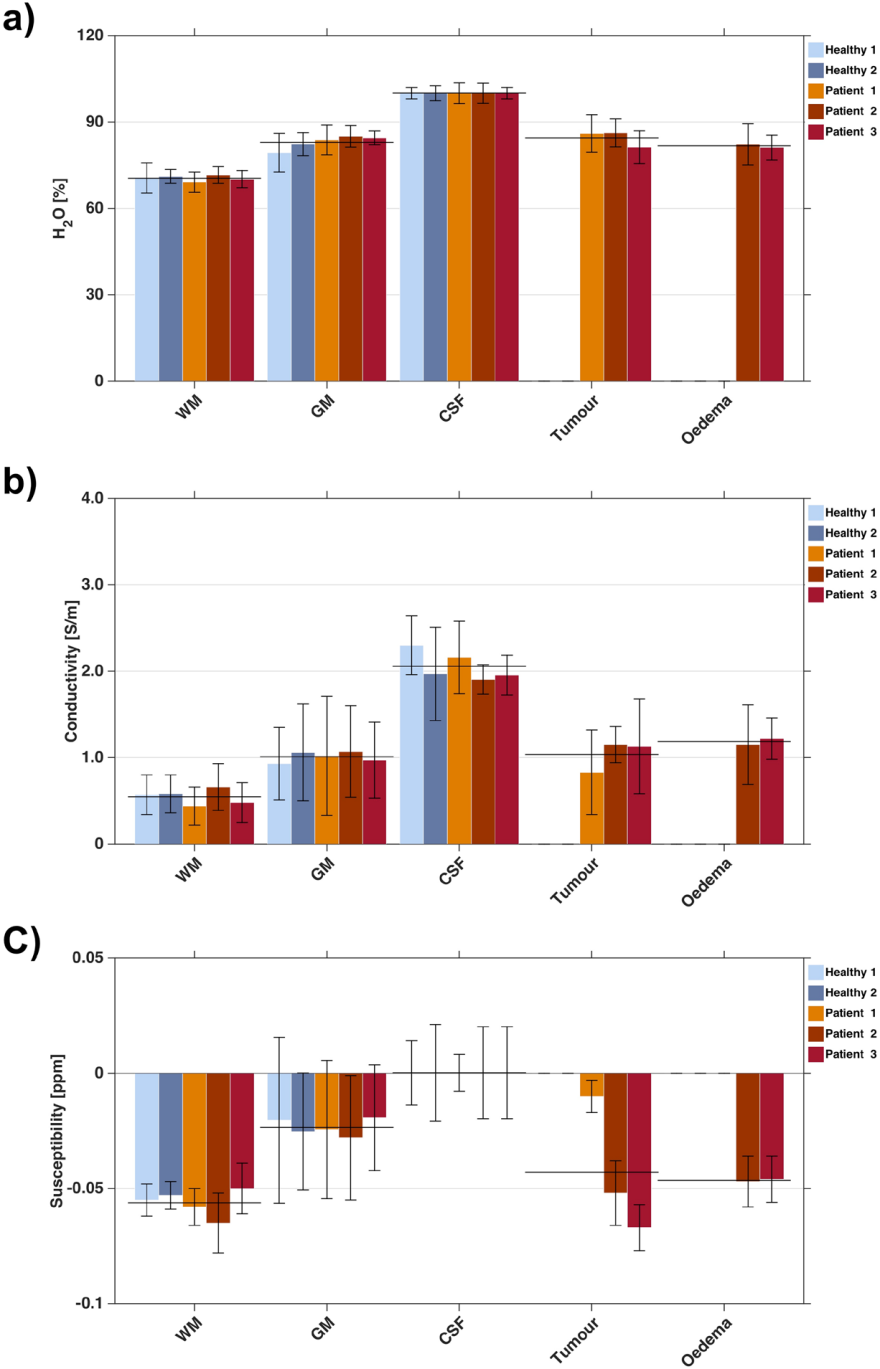
Mean values with standard deviations of (**a**) the water content (**b**) conductivity and (**c**) relative susceptibility with respect to CSF parameters in different segmented tissue groups for five *in vivo* datasets, including two healthy controls and three patients affected by brain tumours. In addition, the overall measured tissue group mean values are indicated.

To investigate the physical changes in abnormal tissues, the averaged values of quantitative parameters and the corresponding standard deviations for WM, GM, CSF, tumour and oedema regions were calculated and are reported in Table 1. For a quick overview, these data were visualised for three quantitative parameters (H_2_O content, QSM, QCM) in Fig. 5. A large variability within a tissue class is visible for QSM values, whereas the QCM values and especially the water content are more narrowly defined for each tissue, whether healthy or affected by tumour. The mean H_2_O content and QCM values in tumour and oedematous areas revealed an obvious increase compared to the surrounding WM, showing similar values to those of cortical GM regions. The contrast and changes caused by pathology in QSM values are more variable. The intra-group variations in WM, GM, and CSF mean values for water content, conductivity and susceptibility were small, suggesting that the underlying physiological properties in those tissues are well regulated. However, for the abnormal brain regions, the amplitude of the changes in the quantitative parameters was considerable. For example, in patient I (meningioma), the mean susceptibility value (relative to CSF) was −0.010 ± 0.007 ppm and the QCM was 0.83 ± 0.49 S/m which differed clearly from patient II (glioblastoma), with −0.052 ± 0.014 ppm and 1.15 ± 0.21 S/m. Water content shows smaller, but better defined changes between healthy and pathological tissue.

## Discussion

A general framework for obtaining quantitative water content, susceptibility and conductivity images in a single acquisition is proposed in this study. This was implemented by exploiting both the magnitude and phase evolution during echo time intervals from a 2D multi-echo GRE sequence. The method is beneficial for *in vivo* MR measurements, especially on patients, where acquisition time needs to be kept to a minimum. This avoids performing separate, sequential measurements for each parameter. Moreover, the method is advantageous for quantitative analysis since it makes co-registration unnecessary.

The proposed method was initially evaluated on a multi-compartment phantom with different concentrations of D_2_O, Gd and NaCl, where the water content, susceptibility and conductivity values increased successively. The reconstructed H_2_O content, QSM and QCM maps of the phantom conformed to the expected behaviours. Two different methods for transmit-receive field inhomogeneity correction were employed separately and compared. The water content values shown in Fig. 2, using both methods, and the corresponding linear regression coefficients (slope = 1.05, r = 0.99) between them, indicate that the methods are both satisfactory for the phantom measurement. In Fig. 2, the 2D susceptibility maps obtained were, overall, in reasonable agreement with the 3D multi-echo protocols (slope = 1.12, r = 0.99). However, compared to the 3D methods, the 2D QSM exhibits a higher standard deviation, as shown in (Fig. 2h,i). Moreover, quantitatively, a discrepancy between the 2D and 3D protocol can be observed in terms of a slope difference. A possible explanation for the discrepancy could be a slice-staggering effect which was occasionally noticed in phase and magnitude images especially at longer echo times^27,28^. This leads to discontinuities in the field and T_2_* values between odd and even slices and variability in the QSM reconstruction which is intrinsically a 3D method. The effect may be due to e.g. heating of the system during the long measurement on phantom. In contrast, the 2D GRE phase based conductivity maps, based to the extrapolation of the phase to zero echo time, showed a similar standard deviation to 3D GRE protocol.

Results obtained using the proposed method in two healthy volunteers confirmed the findings obtained in the phantom experiments. By comparison with the 3D GRE phase-based QSM maps, we can conclude that the tissue contrast in the 2D GRE-based QSM maps is largely preserved. Slice-to-slice inconsistencies were occasionally observed in *in vivo* measurements, where their likely origin is physiological, such as pulsatory motion or respiration. These inconsistencies in tissue phase maps degraded the accuracy and contrast of the QSM reconstruction, as shown by the sagittal slices in the 2D susceptibility maps in Fig. 3c. This problem could be mitigated by using a recently proposed 2D phase processing method, which includes 2D phase unwrapping followed by 2D background phase removal^29^. Although inconsistency between slices was not observed in conductivity reconstruction, the obtained 2D conductivity values were globally slightly higher than 3D conductivity values, as demonstrated by the histogram in Fig. 3. The discrepancy between the two conductivity measurements can be partly attributed to the different effect of physiological motion during the measurement. However, a more detailed analysis would need to be performed in order to accurately determine the source of this difference. Note that the 3D-based GRE results are of course not the ground truth. However, since both conductivity and susceptibility reconstructions are 3D, artefacts resulting from possible slice-related mismatches and offset are avoided despite the longer acquisition time. Hence, 3D data is an excellent reference to “substantiate” the functionality of presented 2D method.

The presented method, in a much faster form, was included in a protocol used in our institution for the examination of patients affected by intracranial tumours. Preliminary results regarding water content and T_2_* results in tumour patients were reported using this fast protocol^8^. The water content maps showed a remarkable enhancement of the signal from abnormal regions, which indicated the presence of oedema – by definition an increase in water content – known to be associated with brain tumours. This was in accordance with the contrast change in the T_1_-weighted MPRAGE and the T_2_-weighted FLAIR. The conductivity of biological tissues is mainly determined by the concentration and mobility of ions. The observed increase in conductivity can be associated with increased sodium ion concentrations within the abnormal areas, including oedema and tumour regions. These findings are in line with a previous study on sodium MR imaging of brain tumours^30^. The biophysical origin of susceptibility contrast is more complex than varying water content and sources of electrical conductivity. It is well known that the susceptibility of GM – especially deep grey matter - is dominated by tissue iron which is mostly stored in ferritin macromolecules^31^. However, in WM, the bulk tissue magnetic susceptibility is substantially affected by myelin, which counteracts the effects of iron^32^. Consequently, in abnormal regions, the observed variation of susceptibility values between WM and cortical GM could be attributed to the internal destruction of myelin or the deposition of ferritin.

A further step in the analysis is to segment the tumour-affected regions into tumour and oedema compartments by applying appropriate thresholds to PET (active tumour tissue) and FLAIR (oedema and tumour) images. For cases II and III, tumours were confirmed as malignant by histological diagnosis, while case I was benign. As demonstrated in Fig. 5, no significant difference between malignant (case II, III) and benign (case I) cases was found for water content values, while conductivity and susceptibility parameters were found to be considerably different in malignant and non-malignant tumours. However, further studies are needed to confirm these findings in a larger patient group. A recent study on electrical conductivity imaging of brain glioma based on MREPT observed significantly increased conductivity values in gliomas, as compared to those of normal brain parenchyma^33^. In addition, higher mean conductivity values were found in the tumour part than in oedema area for the grade IV glioma group. Our observation agrees with the first part of these findings, as shown in Fig. 4, however, no obvious difference was found when the tumour and oedema were compared for conductivity values. This may be caused by the similarity of oedema and tumour signal in the M_0_ weighted images, which are employed to identify local homogeneous regions during the conductivity reconstruction. In this way, the oedema and the tumour were erroneously assigned to one homogeneous region when performing the Laplacian and median filter calculations. This problem could potentially be mitigated by using new reference images which display better contrasts between oedema and tumour regions or by employing new reconstruction strategies free of local homogeneous assumption^34^.

**Figure 6:**
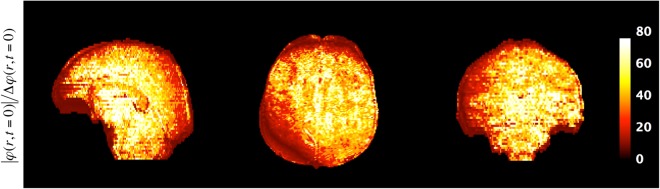
Images of the ratio of fitted zero echo time value to its uncertainty: $|{\rm{\phi }}({{\rm{r}}}_{0},{\rm{TE}}=0)|/{\rm{\Delta }}{\rm{\phi }}({{\rm{r}}}_{0},{\rm{TE}}=0)$ in one representative healthy volunteer. The average value is around 40 within all brain regions.

In this study, we employed a 2D multi-echo GRE to characterise the phase evolution information and derive the RF-induced phase by linear fit extrapolation of this evolution to TE = 0. Generally, the accuracy of the phase estimation at TE = 0 is proportional to the number of echoes involved. However, in some regions, T_2_* decay effects lead to phase values below the noise level for later echoes which prevents further evaluation of the linear phase evolution and, thus, negatively affect the accuracy of the reconstruction. One way to describe the error propagation during the linear regression would be the estimation of the uncertainty of the intercept (extrapolated phase at TE = 0). Maps of the ratio of $|{\rm{\phi }}({{\rm{r}}}_{0},{\rm{TE}}=0)|/{\rm{\Delta }}{\rm{\phi }}({{\rm{r}}}_{0},{\rm{TE}}=0)$ in one representative healthy volunteer were displayed in three orthogonal planes in Fig. 6 during the phase processing. By trying to minimise the uncertainty of the extrapolated phase at TE = 0, we have therefore included a fixed number of 8 echoes in the phase processing. As demonstrated in the Figure, the resulting fitted zero echo phase value is 40 times higher on average compared to the uncertainty which indicates an accurate phase estimation at TE = 0.

Another limitation of the method is that in order to keep T_1_ saturation effects of tissue to a negligible level and to obtain a maximal signal available from the sample, a relative long TR = 10 s is required in conjunction with a flip angle of 90°. Recently, a water content mapping study revealed that the TR can be reduced from 10 s to 5 s, resulting in an acquisition time below 5 min and allowing for an increase in resolution^14^. In order to keep the T_1_ saturation effects at the same level, the flip angle was reduced from 90° to 25°. The very significant loss in SNR was compensated by employing a principal component analysis (PCA)-based noise-reduction method. As a result, the precision and accuracy of the water content maps was shown to be without substantial loss. In the future it is interesting to investigate the accuracy of susceptibility and conductivity mapping with this method, which potentially allows for whole-brain multi-parametric mapping (water content, T_2_*, susceptibility and conductivity mapping) in less than 5 minutes.

## Conclusions

A novel framework for the acquisition of quantitative multi-parametric maps using 2D multi-echo GRE sequence has been demonstrated. Through the reported phantom and healthy control MRI experiments, we have confirmed that the proposed method enables simultaneous mapping of water content, susceptibility and conductivity parameters comparable to reference protocols. The application of this method for quantitative characterisation of brain tumours has been demonstrated in three patients. Signal intensity changes were found in H_2_O content, QSM and QCM parameters, and are the source of the contrast changes in the clinical MR images which are routinely employed for diagnosis. The combined information derived from multi-parametric mapping therefore provides a complex tool, which is different and complementary to FET-PET, for the analysis and treatment of pathologies within the time frame of a standard clinical protocol acquisition.

## Data Availability

The datasets acquired and processed during the current study are not publicly available due to restrictions in ethical approval, however are available from the corresponding author on reasonable request.
